# Striatopallidal neurons control avoidance behavior in exploratory tasks

**DOI:** 10.1038/s41380-018-0051-3

**Published:** 2018-04-25

**Authors:** Kimberly H. LeBlanc, Tanisha D. London, Ilona Szczot, Miriam E. Bocarsly, Danielle M. Friend, Katrina P. Nguyen, Marda M. Mengesha, Marcelo Rubinstein, Veronica A. Alvarez, Alexxai V. Kravitz

**Affiliations:** 1 National Institute of Diabetes and Digestive and Kidney Diseases, National Institutes of Health, Bethesda, MD 20892 USA; 2 National Institute on Alcohol Abuse and Alcoholism, National Institutes of Health, Bethesda, MD 20892 USA; 3 Instituto de Investigaciones en Ingeniería Genética y Biología Molecular, CONICET, Buenos Aires, C1428ADN Argentina; 4 FCEN, Universidad de Buenos Aires, Buenos Aires, C1428EGA Argentina; 5 Department of Molecular and Integrative Physiology, University of Michigan Medical School, Ann Arbor, MI 48109 USA; 6 National Institute on Drug Abuse, National Institutes of Health, Bethesda, MD 20892 USA

**Keywords:** Neuroscience, Physiology

## Abstract

The dorsal striatum has been linked to decision-making under conflict, but the mechanism by which striatal neurons contribute to approach-avoidance conflicts remains unclear. We hypothesized that striatopallidal dopamine D2 receptor (D2R)-expressing neurons promote avoidance, and tested this hypothesis in two exploratory approach-avoidance conflict paradigms in mice: the elevated zero maze and open field. Genetic elimination of D2Rs on striatopallidal neurons (iMSNs), but not other neural populations, increased avoidance of the open areas in both tasks, in a manner that was dissociable from global changes in movement. Population calcium activity of dorsomedial iMSNs was disrupted in mice lacking D2Rs on iMSNs, suggesting that disrupted output of iMSNs contributes to heightened avoidance behavior. Consistently, artificial disruption of iMSN output with optogenetic stimulation heightened avoidance of open areas of these tasks, while inhibition of iMSN output reduced avoidance. We conclude that dorsomedial striatal iMSNs control approach-avoidance conflicts in exploratory tasks, and highlight this neural population as a potential target for reducing avoidance in anxiety disorders.

## Introduction

Animals often make decisions that require balancing potential risks against rewards in uncertain environments. A framework known as reinforcement sensitivity theory (RST) frames these decisions as interactions between three competing systems: (1) a behavioral activation system (BAS) that reacts to salient stimuli and drives approach; (2) a fight, flight, freeze system (FFFS) that controls behavioral responses to threatening or potentially aversive stimuli, and (3) a behavioral inhibition system (BIS) that is activated by conflict or competition between the BAS and FFFS [1, 2]. During such conflict, the BIS delays actions so additional information can accrue before selecting a behavior, an adaptive strategy known as risk assessment [3, 4]. Imbalances between these systems are hypothesized to underlie several neuropsychiatric disorders. For example, people with anxiety disorders often ruminate excessively over decisions, which may be a maladaptive form of risk assessment [3]. Anxiolytic drugs have long been hypothesized to tamp down the BIS [5], and are most effective at modifying animal behavior in tasks that invoke approach-avoidance conflict [1, 6]. Thus, understanding the neural circuitry that underlies the BIS may have implications for treating avoidance behavior in anxiety disorders.

The neural circuitry of the BIS has often been attributed to canonical fear pathways including the prefrontal cortex, hypothalamus, amygdala, and periaqueductal gray [7, 8]. While the striatum is not often linked to the BIS, it is well situated to coordinate information within this circuitry, receiving strong excitatory innervation from the prefrontal cortex and amygdala, and having downstream projections to the pallidum, hypothalamus, and midbrain [9–15]. The dopamine system and the ventral striatum have been implicated in approach behavior [16–19], as well as aversion and negative affect [20–24]. Fewer studies have focused on the dorsal striatum’s contributions to approach and avoidance; however, lesions of the dorsal striatum impair conditioned emotional responses, conditioned freezing, and passive and active avoidance [25–29]. More recently, the dorsal striatum has also been implicated in balancing risk and reward and making decisions under conflict [30–33], suggesting that the striatum’s role in the BIS and approach-avoidance conflict may be underappreciated [7, 8].

The dorsal striatum contains two classes of projection neurons, direct and indirect pathway medium spiny neurons (dMSNs and striatopallidal neuron (siMSNs)), which differ in both their projection targets and dopamine receptor subtypes [34]. Whereas activation of dMSNs facilitates approach [35–37], iMSNs are implicated in risk aversion and avoidance [35, 36, 38, 39]. iMSNs express the dopamine D2 receptor (D2R), and genetic polymorphisms in D2R gene expression have been linked to avoidance behavior in social phobia [40], social dysfunction [41], alcoholism associated anxiety [42], and anorexia [43]. Polymorphisms in the D2R gene have also been linked to avoidance behavior in people without neuropsychiatric disorders [44]. Based on this evidence, we hypothesized that D2R-expressing dorsal striatal neurons contribute to avoidance behaviors in approach-avoidance conflict scenarios and are critical neural component of the BIS.

Multiple striatal cell-types express D2Rs, including iMSNs, dopaminergic terminals, and cholinergic interneurons [34, 45–49]. Since many investigations of striatal D2R function in risk aversion and avoidance behavior used pharmacological or neuroimaging techniques that cannot discriminated between the different neural populations in the striatum that express D2Rs, it is unclear which population of D2R-expressing striatal cell-types contribute to avoidance behaviors. To assess this, we selectively removed D2Rs from iMSNs (iMSN-Drd2KO [50]), cholinergic interneurons (CIN-Drd2KO), or dopamine neurons (DAT-Drd2KO [45]), and tested whether these conditional mutant mice exhibited changes in avoidance behavior in the elevated zero maze and the open field. These tests were chosen as they allow for repeated conflicts between novel exploration and potential threats without the use of strong aversive stimuli like electric shock (i.e.: Geller-Seifter and Vogel conflict tasks). In addition, these tasks can dissociate changes in overall activity levels from changes in risky behavior (time spent in open areas), which is critical for our experiments, since strong activation of iMSNs interferes with movement [37, 39, 51].

iMSN-Drd2KO mice, but not CIN-Drd2KO or DAT-Drd2KO mice, avoided the open areas in both tasks, independent of global changes in movement levels. We focused on the dorsomedial striatum (DMS) for additional study, based its role in goal-directed behavior [52], and links between DMS iMSNs and aversion [36]. Population calcium recordings of DMS iMSNs [53], revealed that iMSN-Drd2KO mice had disorganized iMSN activity around movements into the open areas of both tasks, potentially contributing to the reduction in movements into these areas. Disrupting iMSN output via optogenetic stimulation also caused mice to avoid the open areas of these tasks, supporting the conclusion that aberrant output of iMSNs can promote avoidance. Finally, inhibition of iMSNs in the DMS via designer receptors [54] reduced avoidance in both WT and iMSN-Drd2KO mice, highlighting the potential of iMSNs as a target for reducing aberrant avoidance in anxiety disorders.

## Materials and methods

### Experimental model and subject details

Mice (all C57/BL6 background, p60–180) were housed in a modified 12 h light/dark cycle kept at 23–25 °C. Food and water were given ad libitum, and cages were changed weekly or bi-weekly. All were of normal weight and immune status. All animal studies were approved by the National Institute of Diabetes and Digestive and Kidney Diseases (NIDDK)/National Institutes of Health (NIH) Animal Care and Use Committee.

#### KO experiments

Cre lines (A2A-cre, DAT-cre, and ChAT-cre) were generated by the GENSAT project [68]. Drd2KO mice were generated by crossing Drd2loxP/loxP mice [45] which carry the conditional Drd2 alleles in homozygosity, with bi-transgenic mice that were heterozygous for the Drd2lox allele, and also contained: A2A-Cre+/− (iMSN-Drd2KO, *n* = 11), ChaT-Cre +/− (CIN-Drd2KO, *n* = 12), or DAT-Cre +/− (DAT-Drd2KO, *n* = 10), which express Cre recombinase in heterozygosity under the respective promoter. Control littermates were Cre −/− littermates from these crosses (iMSN-Drd2-WT (*n* = 14), CIN_Drd2-WT (*n* = 9), DAT-Drd2-WT (*n* = 10)), which contained intact D2Rs. Both male and female mice were included in these experiments, and mice were group housed as littermates. There was no evidence of sex differences in our behavioral measurements and therefore data from males and females were combined. Experimenter was blinded to the genotype of the mice.

#### Optogenetic, fiber photometry, and designer receptor exclusively activated by designer drugs (DREADD) experiments

Experiments expressing ChR2, GCaMP6s, or DREADD receptors used male mice, which were singly housed after surgery to protect the cranial implants. No mice had prior drug exposures other than those associated with surgery and post-operative care. These experiments used the A2A-Cre line described in the section above. The freely-moving optogenetic experiments included A2A-Cre+ mice expressing ChR2 (iMSN-ChR2, *n* = 13) and A2A-Cre −/− littermates (*n* = 21) that received the ChR2 virus as controls. The transition zone stimulation optogenetic experiment used iMSN-ChR2 mice (*n* = 9). Fiber photometry experiments used both A2A-Cre+ mice (*n* = 9) and iMSN-Drd2KO mice (*n* = 7). KOR-DREADD experiments used A2A-Cre+ mice (*n* = 15) and iMSN-Drd2KO mice (*n* = 6).

### Viral expression and optical implants

Mice were 6–8 weeks old at the time of surgery. Anesthesia was induced with 2–3% isoflurane (vol/vol) and maintained with 0.5–1.5% isoflurane through a nose cone mounted on a stereotaxic apparatus (Stoelting Co.). A 5 μl Hamilton syringe with a 33-gauge metal needle was used to infuse the virus with a mircrosyringe pump (KdScientific) at a rate of 50 nl/min over 10 min. See Table 1 for viral vectors used. All stereotaxic coordinates were in relation to bregma for dorsomedial striatum (DMS): anterior-posterior, 0.5 mm; medial-lateral, +/−1.5 mm; dorsal-ventral, −2.8 mm. Following infusion, the needle was kept at the injection site for 5 min then slowly withdrawn. For optogenetic experiments, fiber optic assemblies consisting of a plastic mount containing two fibers (200-μm core and 220-μm cladding) mounted in 1.25-mm zirconia ferrules were implanted in the DMS (anterior-posterior, 0.5 mm; medial-lateral, +/−1.5 mm; dorsal-ventral, −2.6 mm) of each experimental mouse. Cannulas were secured to the skull using a base layer of adhesive dental cement (C&B Metabond; Parkell) followed by a second layer of cranioplastic cement (Stoelting Co). For fiber photometry, unilateral optic-fiber cannulas were used (fiber: core = 200 μm; 0.48 NA; M3 thread titanium receptacle; Doric Lenses).

**Table 1:** Viral information and sources

Viral vector	Abbreviation	Serotype/titer	Volume /side	Time post surgery for expression	Source
rAAV-Ef1aDIO-hChR2(H134R)	ChR2	serotype AAV8, titer = of 8×10^12^	500 nl	At least 2 weeks	Penn vector core
Syn-DIO-hKORD-IRESmCit-WPRE	KOR-DREADD	serotype AAV8, titer = 1.2×10^13^	500 nl	At least 8 weeks	UNC vector core
AAVdj-EF1α-DIO-GCaMP6s	GCamp6s	serotype AAVDJ, titer = 3.1 × 10^12^	500 nl	At least 8 weeks	UNC vector core
AAV8-Hsyn-GFP	GFP	Serotype AAV8	500 nl	At least 8 weeks	UNC vector core

### Behavioral testing

Behavioral tests occurred during the light cycle. Mice were either housed in the behavioral testing area or moved to a behavioral suite and allowed to acclimate for at least 30 min. Mice were handled for at least 3 days prior to testing. Behavioral measurements were collected using Noldus Ethovision software (Noldus Information Technologies, NED) and analyzed with customized Python scripts (https://github.com/KravitzLab).

Open field locomotion was measured in Noldus PhenoTyper 3000 (30 cm × 30 cm x 35 cm, length × width × height) polycarbonate chambers or in a large (40 cm × 40 cm × 40 cm) acrylic/PVC chamber. The center area was defined as 65% of the open field arena. The zero maze was 57 cm in diameter and 34 cm high off the ground, and consisted of two “open” arms (7 cm wide) without walls, and two “closed” arms that were enclosed by IR-transparent plastic that allowed for IR-video tracking within the closed arms. Mice started in the open arm of the elevated zero maze, and sessions began once the mouse had moved into the closed arm. In our fiber photometry experiment, we observed a high frequency of transients occurring when the mouse was at the edge of the closed arm, facing the open arm. To better categorize this activity, we defined the four areas at the transition of open and closed as the mouse being in a “transition zone”. This was quantitatively defined as a 30-degree arc centered on the boundary between the open and closed arm, thereby allocating equal total area (120 degrees each) to the “open”, “closed”, and “transition” zones. We used the same parameters for the transition zone stimulation experiment.

For KO experiments, zero maze and open field sessions lasted 10 min. For photometry experiments, mice were recorded for 30 min on both the zero-maze and the open field. For optogenetic experiments, mice were placed in the task for either a 1-min or 5-min baseline period followed by 5 cycles of 1 min on, 1 min off stimulation at 0.1–0.2 mW. For DREADD experiments, animals first acclimated for 1 h prior to injection, after which they were injected with either Salvinorin B (17 mg/kg) or DMSO (vehicle). Measurements began 20 min post-injection and lasted 10 min. In the transition zone stimulation experiment, mice received a two-second, 200 µW burst of stimulation when they moved from the closed arm to the transition zone or from the open arm into the transition zone. Mice were required to remain in the transition zone for at least 0.5 s for the trial to start and the stimulation to be delivered. Stimulation trials were interspersed with no stimulation control trials, in which the mouse would enter the transition zone but would not receive stimulation. Stimulation and no stimulation trials were delivered pseudo-randomly. There was a total of 40 trials, 10 each of closed to transition zone stimulation trials, closed to transition zone no stimulation trials, open to transition zone trials, and open to transition zone no stimulation trials.

Movements were defined as periods in which the mouse was moving at 2 cm/s or faster for at least 500 ms. Movements in the sub-regions of the apparatus (open or closed arms of the zero maze, center or surround of the open field) were derived as a subset of total movements, and included both movements that resulted in entering the sub-region for at least 500 ms during a movement bout as well as movements entirely within that sub-region. Thus, if a mouse spent at least 500 ms in the open arm during a movement, or if the mouse began and ended a movement entirely within the open arm, this movement was considered a movement in the open arm.

### Fiber photometry

Mice were connected for fiber photometry with a single optic fiber (core = 200 μm; 0.48 NA; M3 connector; Doric Lenses). Blue light (475 nm LED, Plexon Inc) was modulated with an 80 Hz sinusoid waveform from a function generator (B&K Precision, model 4054B) and delivered to the brain. The average power of this modulated illumination was 20–40 μW. The emitted green fluorescence passed through a dichroic mirror and 505–535 nm cut filter (FMC4 port minicube, Doric Lenses) and was detected with a femtowatt silicon photoreceiver (Model 2151, Newport). Analog signals from the detector were then amplified and recorded with a digital acquisition system (Omniplex, Plexon Inc). Synchronized videos were recorded simultaneously via Ethovision XT (Noldus Information Technologies, NED).

Raw signals were demodulated and converted to df/f with custom Python scripts that were executed in Neuroexplorer V5 (scripts available at: https://github.com/KravitzLab, Figure S3). These scripts demodulated the raw signal by returning power at 79–81 Hz, using the spectrogram analysis in Neuroexplorer v5. Next, the demodulated signal was transformed into df/f using a rolling average of 1-min around each data point as *f*, and normalizing each data point *f*_*n*_ with the formula (*f*_*n*_-*f*)/*f*.

Photometry signals were exported as average peri-event time histograms around these events, and specific behavioral periods were extracted for analysis. These periods were: baseline (a 5 s period beginning 15 s before the entry into the zone), pre-movement (a 5 s period beginning 8 s before the entry into the zone) and movement (a 1 s period beginning at the peak of the movement). Average d*f*/*f* in each period was generated for each mouse, and these averages were entered into a RM-ANOVA to detect effects of behavioral period on photometry signals in each group.

### Histology

Brains were removed and post-fixed in 10% formalin. Brains were transferred to 30% sucrose in PBS for 2–3 days, until saturated, and then sectioned at 40 µM on a freezing microtome (Leica). Sections were counter stained with DAPI and mounted on slides for imaging with a slide-scanning microscope (Olympus VS120). After scanning, fluorescence areas were outlined in ImageJ (https://imagej.nih.gov/ij/) and positioned over a corresponding atlas section in Illustrator (Adobe).

### Quantification and statistical analyses

Video or mouse behavior was processed and quantified with Ethovision XT (Noldus Information Technologies). Data was organized in Microsoft Excel. Statistical comparisons were made in Graphpad Prism version 7, via ANOVAs and *t*-tests where specified. Randomizations were performed for counter-balanced experiments by alternating mice based on mouse number. All comparisons met the assumptions of the test used, including similar variance between groups being compared.

## Results

### Avoidance behavior in the zero-maze and open-field tasks

We first investigated how specific behavioral features of wildtype mice (25 males, 8 females) correlated with the time they spent in the open areas of these tasks, using a multiple regression analysis. The features tested were (1) speed while moving, (2) average duration of movement, (3) total number of movements, (4) total distance moved, and (5) number of movements into the open area of each task. The regression model was significant for both zero maze (*F*(5, 27) = 28.5, *p* < 0.0001) and open field (*F*(5, 27) = 32.7, *p* < 0.0001), and indicated that ~85% of the variance of the time spent in the open areas was captured by these five behavioral variables (zero maze *R*^2^ = 0.84, open field *R*^2^ = 0.86). On the zero maze, the strongest single behavioral correlate was the number of movements into the open arms of the maze (Figure S1F, *p* < 0.0001, full statistics in Table S1), consistent with the view that the zero-maze evaluates the balance between approach and avoidance, more so than global differences in activity [7]. The average duration of movements also formed a slight but significant (*p* = 0.02, Figure S1D) correlation with time in the open arms, indicating there may be a link between the mechanics of individual movements and approach behavior in this task. Speed while moving, total number of movements, and total distance did not correlate significantly with time in the open arms (all *p* > 0.10, Figure S1B, C, E), indicating that overall activity level is not a strong correlate of approach behavior in this task. For the open field, the strongest correlate was also the number of movements into the center (Figure S1L, *p* < 0.0001), and no other movement feature formed a significant correlation with time in the center (all *p* > 0.15, Figure S1G-K). Together these results demonstrate that the percent of time spent in risky areas of these tasks reflects decisions to initiate movements into these regions and not global metrics of activity.

### D2Rs on indirect pathway neurons control approach-avoidance balance

**Fig. 1:**
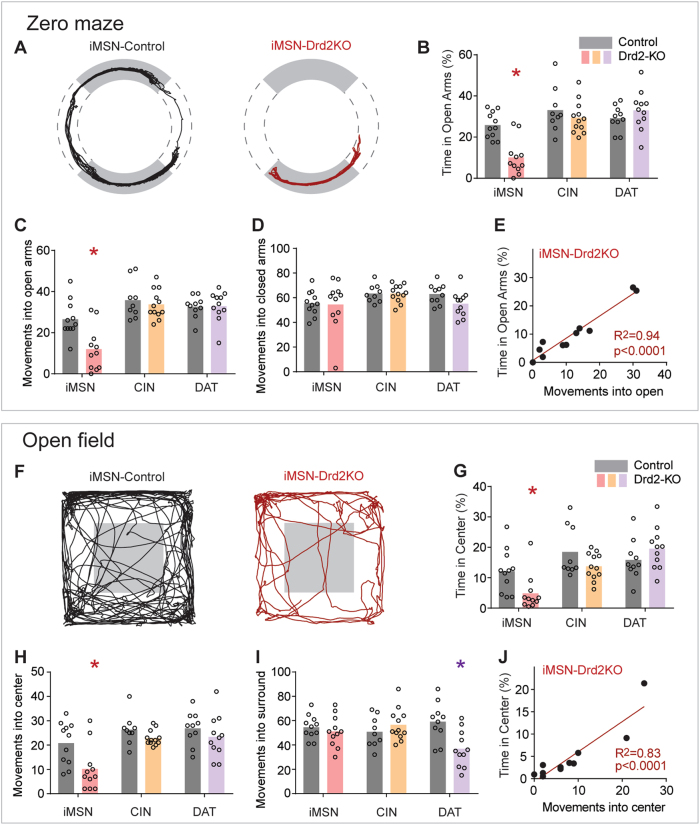
Removing D2Rs from iMSNs promoted avoidance in exploratory tasks. **a** Path plots for control and iMSN-Drd2KO mouse in the zero maze. **b** Time in open arms, **c** movements into the open arms, and **d** movements into the closed arms for iMSN-Drd2KO, CIN-Drd2KO, and DAT-Drd2KO mice. **e** Correlation between time in open arms and number of movements into the open for iMSN-Drd2KO mice. **f**–**j** Same presentation as (**a**–**e**), but for open field data. Circles reflect individual mice, lines are linear regressions. *s denote significance between control and iMSN-Drd2KO by Sidak’s post hoc test following 2-way ANOVA

To identify the contribution of different populations of striatal D2Rs to avoidance behavior in these tasks, we used a breeding strategy that generated mice lacking D2Rs in striatal indirect pathway medium spiny neurons (iMSN-Drd2KO, *n* = 11), cholinergic interneurons (CIN-Drd2KO, *n* = 12), or dopaminergic neurons (DAT-Drd2KO, *n* = 10 Figure S2A), and tested these mice and their littermate controls (iMSN-Drd2-WT, *n* = 11, CIN-Drd2-WT, *n* = 9, DAT-Drd2-WT, *n* = 10) on the zero maze and open field. iMSN-Drd2KO mice, but not the other two lines, spent less time in both the open arms of the zero-maze and the center of the open field compared to littermate controls (Fig. 1a,b, 2-way ANOVAs, Zero-maze interaction: *F* (2, 57) = 7.6, *p* < 0.002, Open field interaction: *F* (2, 57) = 4, *p* < 0.03, Sidak’s post hoc tests both *p* < 0.05). This was associated with fewer movements into the open arms and center in iMSN-Drd2KO mice (Fig. 1c,h, 2-way ANOVA with Sidak’s post hoc tests *p* < 0.05), but not into the closed arms or surround of the open field (Fig. 1d,i, *p* > 0.26 for all). As with wildtype mice, entries into the open areas also correlated strongly with the total time spent in these areas in the iMSN-Drd2KO mice (*R*^2 ^> 0.94, *p* < 0.0001 for zero maze and *R*^2^ > 0.83, *p* < 0.0001 for open field, Fig. 1e,j). These correlations also held when controlling for total distance moved (*R*^2^ = 0.68, *p* < 0.005 for zero maze, *R*^2^ = 0.90, *p* < 0.0001 for open field). These results demonstrate that D2Rs on iMSNs, but not cholinergic neurons or dopamine neurons, control avoidance behavior in both tasks, independently of differences in overall activity levels.

To further evaluate the above claim, we examined finer aspects of movement in all three lines of mice. iMSN-Drd2KO mice had lower average velocity in both tasks, with slightly slower speeds while moving into both open and closed areas of the task, as well as shorter durations of movements (RM ANOVAs followed by Sidak’s post hoc: *p* < 0.01 for all, Figure S2), consistent with prior literature [50] and with movement patterns during approach-avoidance conflict [8]. DAT-Drd2KO mice had longer durations of movement in both tasks, and had higher average velocity and velocity of movement in the open field (Sidak’s post hoc: *p* < 0.005, Figure S2), consistent with prior reports on these mice [45]. There was no effect of knocking out the D2R on cholinergic interneurons on any behavioral measure (all *p* > 0.26, full statistics in Table S1).

### Population calcium activity of iMSNs was disrupted in iMSN-Drd2KO mice

**Fig. 2:**
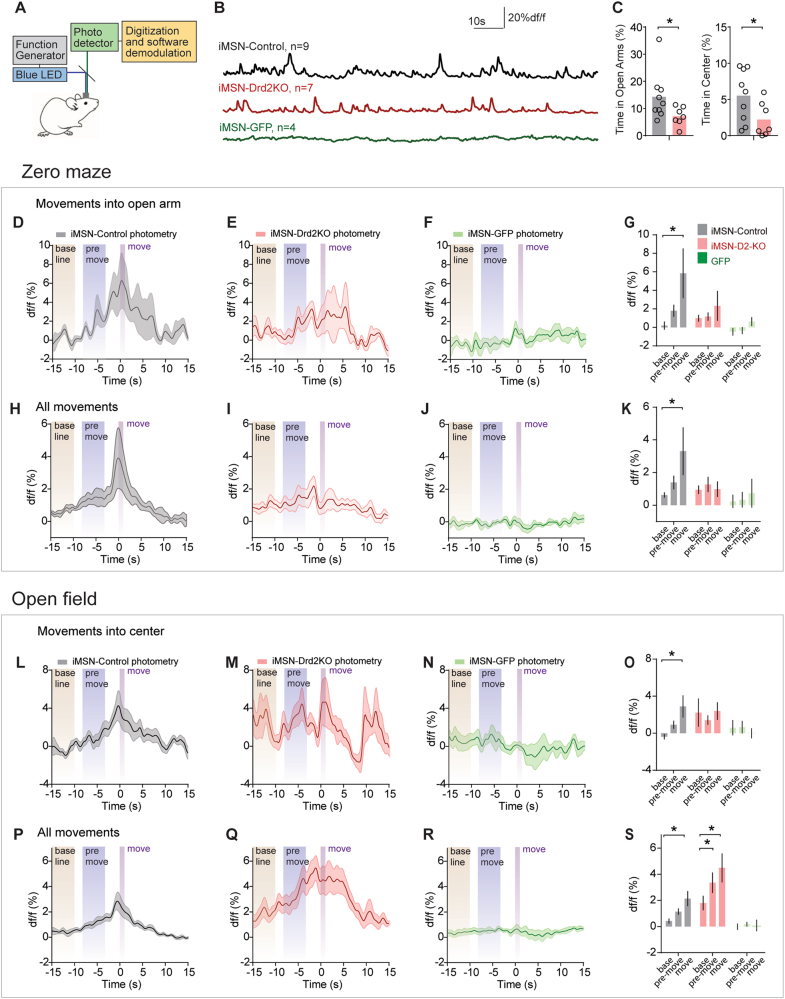
Population calcium signaling in iMSN-Control and iMSN-Drd2KO mice. **a** Schematic of photometry system. **b** Example traces from iMSN-Control, iMSN-Drd2KO, and iMSN-GFP mice. **c** Time spent in open arms and center for iMSN-Control and iMSN-Drd2KO mice. **d**–**f** Average photometry recordings around movements into the open arms for iMSN-Control, iMSN-Drd2KO, and iMSN-GFP mice. Periods for ANOVA analysis noted in vertical bars. **g** Average d*f*/*f* for baseline, pre-movement, and movement periods. **h**–**k** Same as (**d**–**g**) but for all movements. **l**–**s** Same data presentation as (**d**–**k**) but for open field. *s denote significance between control and iMSN-Drd2KO by Sidak’s post hoc test following two-way ANOVA

To gain insight into why removing D2Rs from iMSNs increased avoidance of the open areas of these tasks, we expressed GCaMP6s [55] in iMSNs in the dorsomedial striatum of control (A2A-cre, *n* = 9) and iMSN-Drd2KO (*n* = 7) mice, and recorded population calcium signals from each group (Fig. 2a,b, S3, histology in Figure S9). iMSN-Drd2KO mice spent less time in the open arms and center than iMSN-Control mice, as in above experiments (Fig. 2c, one-tailed *t*-tests, both *p* < 0.05). We examined fluorescence in three defined periods: baseline (a 5 s period beginning 15 s before the mouse entered the open arms or center), pre-movement (a 5 s period beginning 8 s before the mouse entered these zones) and movement (a 1 s period directly after the mouse entered these zone, Fig. 2d). On average, control mice exhibited ~5–10% increases in calcium activity during movements into the open arms or center, relative to baseline (Figure D, L). In contrast, iMSN-Drd2KO mice did not exhibit a significant increase during these periods (Fig. 2e,m). For the zero maze, post hoc tests (following a RM-ANOVA) revealed a significant difference in activity between baseline and movement in the iMSN-Control mice (*p* < 0.005), but not the iMSN-Drd2KO mice (Fig. 2g, *p* = 0.46). Similar results were found for the open field, where post hoc tests revealed a significant difference in fluorescence between baseline and movement in the iMSN-Control (*p* < 0.005) but not the iMSN-Drd2KO mice (Fig. 2o, *p* = 0.90). Importantly, no significant modulations in calcium activity were observed in iMSN-GFP mice (Fig. 2f,n, all *p* > 0.6 in post hoc tests). Despite the reductions in average velocity in the iMSN-Drd2KO mice [50], velocity during these tasks movements was not significantly different in these mice (Figure S4G, O, RM-ANOVA *p* > 0.78 for both). Therefore, the disruption in temporal patterning of iMSN calcium activity was not attributable to differences in movement velocity.

We next examined activity around all movements, regardless of where they occurred in these tasks, to determine whether the differences in calcium signaling were specific to movements into the open areas. For the zero-maze, we observed a similar pattern as with open-arm movements (Fig. 2h–j). Post hoc tests (following RM-ANOVA) revealed a significant difference in activity between baseline and movement in the iMSN-Control mice (*p* < 0.001), but not the iMSN-Drd2KO mice (Fig. 2g, *p* = 0.97). For open field, fluorescence increased significantly between baseline and movement in both iMSN-Control and iMSN-Drd2KO mice (both *p* < 0.001), but also between baseline and pre-movement (*p* < 0.01) in the iMSN-Drd2KO mice (Fig. 2s). GFP expressing mice (*n* = 4) did not exhibit any significant modulation in activity around movements in either task (Fig. 2f,j,n,r, all *p* > 0.6 in post hoc tests). There was again no difference in the velocity of movements on the zero maze (Figure S4K, *p* = 0.57), although there was a difference on the open field (Figure S4S, *p* < 0.01).

Finally, we analyzed the distribution of *peak* activity levels during the movement period between iMSN-Control and iMSN-Drd2-KO mice, to see if there was a between-genotype difference in this measure. We did not detect any significant difference between the two genotypes (Kolmogorov**-**Smirnov tests, *p* < 0.27 for both tasks), which may reflect the high level of variance in fluorescence during movement across mice (Fig. 2k,s). We conclude that the loss of D2Rs in iMSNs is associated with disrupted temporal activity patterns of iMSNs around both “risky” movements into the open areas of the task, and also more generally around movements in these tasks.

### Stimulation of iMSNs promoted avoidance

As iMSN-Drd2KO mice exhibited disorganized calcium activity around movements in these tasks, we tested whether artificially disrupting the output of iMSNs with optogenetic stimulation would also increase avoidance behavior in these tasks. Moderate levels (~1 mW) of iMSN stimulation induces motor freezing [51], which would preclude investigation in exploratory tasks. We therefore first characterized a dose-response curve to determine levels of stimulation that had minimal effects on movement (Figure S5A). We virally expressed channelrhodopsin-2 (ChR2) in iMSNs using a cre-dependent strategy and stimulated mice (*n* = 8) at eight intensities (0, 50 µW, 100 µW, 250 µW, 500 µW, 1 mW, 1.5 mW, and 2 mW, run in a randomized design). We observed decreases in distance and frequency of movement even at the lowest intensities (Figure S5B-C). We used 100–200 µW for the remainder of our optogenetic experiments as this reliably caused behavioral changes without inducing excessive freezing.

**Fig. 3:**
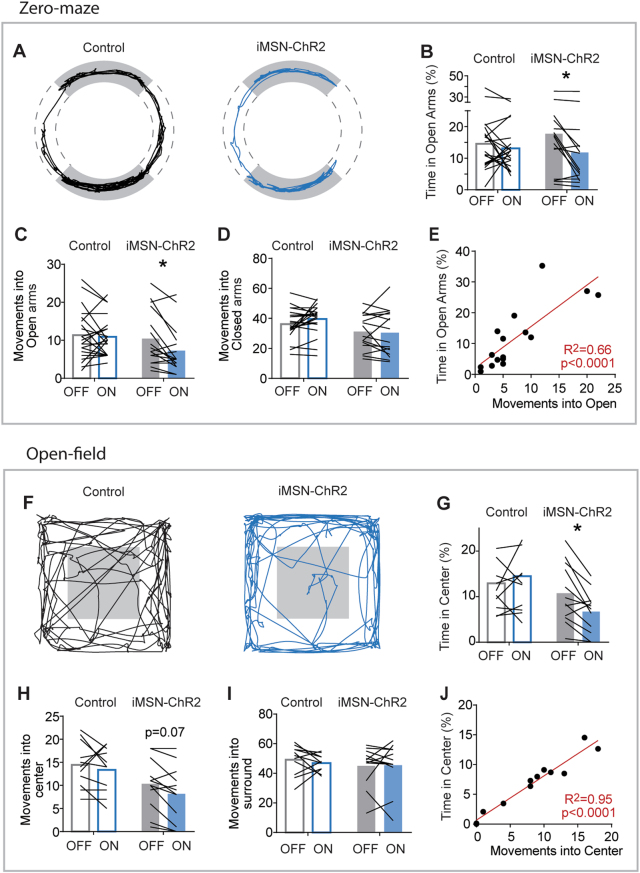
Optogenetic stimulation of iMSNs promoted avoidance of open areas. **a** Example movement traces on zero maze during optogenetic stimulation. **b** Time in open arms, **c** movements into the open arms, and **d** movements into the closed arms for control and iMSN-ChR2 mice. **e** Correlation between time in open arms and number of movements into the open during LED ON for iMSN-ChR2 mice. **f**–**j** Same data format as (**a**–**e**) but for open field. Black lines reflect paired comparison for individual mice, red lines are linear regressions. *s denote significance between control and iMSN-Drd2KO by Sidak’s post hoc test following two-way ANOVA

In a new group of mice (iMSN-Control *n* = 21, iMSN-ChR2 *n* = 16), we stimulated iMSNs in an alternating design (five presentations each of 1-min ON, 1-min OFF, Figure S6). All mice were run in zero maze, while 11 iMSN-Control and 12 iMSN-ChR2 mice were run in open field. Consistent with our hypothesis, low-power stimulation of iMSNs caused mice to spend less time in the open arms of the zero maze and the center of the open-field (2-way ANOVA with Sidak’s post hoc: *p* < 0.02 for both, Fig. 3a,b, f,g), which was associated with fewer movements into open arms of the zero maze (Sidak’s post hoc, *p* < 0.02, Fig. 3c), and a trend towards this for open field (*p* = 0.07, Fig. 3h). Interestingly, we did not observe any significant effect of ChR2 stimulation on the number of movements into the closed arms or surround of open field (both *p* > 0.9, Fig. 3d,i). Again, strong correlations were observed between the number of movements into open areas and the total time spent in these areas during ChR2 stimulation (*R*^2^ > 0.66, *p* < 0.0001 for zero maze, Fig. 3e, *R*^2^ > 0.94, *p* < 0.0001 for open field, Fig. 3j), which remained significant when controlling for total distance moved (*R*^2^ > 0.66, *p* < 0.0001 for zero maze, *R*^2^ > 0.86, *p* < 0.0001 for open field). We analyzed the minute-by-minute data to see if the effect of the stimulation recovered between trials, but did not observe a statistically quantifiable recovery between stimulation trials (Figure S6). An analysis of finer features of movement largely recapitulated what was found in iMSN-Drd2KO mice: a decrease in average velocity and movement duration during stimulation in both tasks (2-way ANOVA with Sidak’s post hoc in ChR2 expressing mice: *p* < 0.05 for both tasks, Figure S5D, H, I, M), without significant changes in velocity of movements (Figure S3E, F, J, K, Sidak’s *p* > 0.05, full statistics in Table S1). These results demonstrate that low power stimulation of iMSNs facilitates avoidance of open areas in these tasks and provides a potential mechanism by which loss of D2Rs on iMSNs increases avoidance—by disrupting iMSN output.

### Stimulation of iMSNs near open areas promoted avoidance

**Fig. 4:**
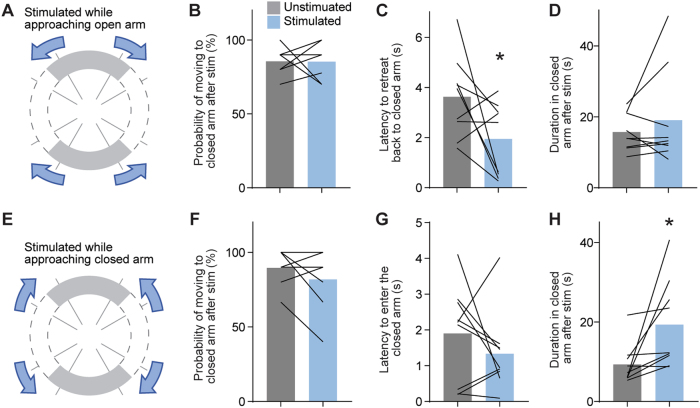
Effect of brief optogenetic stimulation at transition zones on behavior in zero maze. **a** Schematic of stimulation while moving from the closed arm to the transition zone. **b** Probability of movements into the closed arm for stimulated and unstimulated trials. **c** Latency to leave the transition zone following stimulation. **d** Duration in the closed arms following stimulation. **e**–**h** Same data format as (**a**–**d)**, but for stimulation while moving from open to transition. Black lines reflect paired comparison for individual mice. *’s indicate significance with paired 1-tailed *t*-tests

To specifically alter iMSN activity around movements, we conducted “closed-loop” optogenetic experiment in which we delivered brief (2 s at 200 μW) stimulation to iMSN-ChR2 mice (new mice, *n* = 9) when they approached the boundary between the open and closed arms of zero maze. This stimulation was delivered on ten trials when mice approached the open arm and ten trials when they approached the closed arm (Fig. 4a,e). These trials were interspersed with control trials in which no stimulation was delivered. The most common response following an approach towards the open arm was a retreat back to the closed arm, regardless of stimulation state (Fig. 4b). However, mice retreated to the closed arm significantly faster in stimulated trials (Fig. 4c, one-tailed paired *t*-test, *p* < 0.05). Importantly, this effect was counter to the inhibitory effect of iMSN stimulation on movement [51], demonstrating that avoidance behavior can be driven by iMSN stimulation independently of changes in movement. We performed a similar stimulation paradigm on these mice as they moved from the open arm towards the closed arm (Fig. 4e). Again, mice most commonly entered the closed arm following this approach, regardless of the stimulation state (Fig. 4f). In this case, mice remained in the closed arm longer following the stimulated trials (Fig. 4h, one-tailed paired *t*-test, *p* < 0.02). We conclude that short (2 s) bursts of low-power iMSN stimulation can promote avoidance of open areas in these tasks.

### Inhibition of iMSNs reduced avoidance in exploratory tasks

**Fig. 5:**
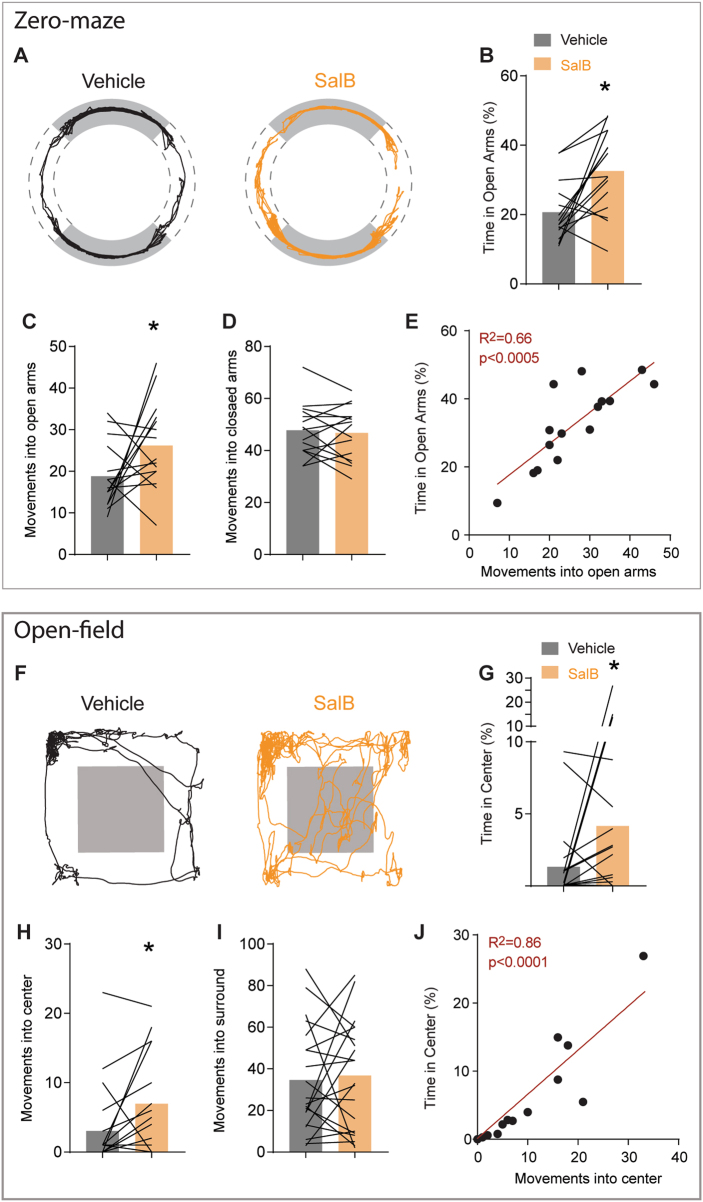
KOR DREADD inhibition of iMSNs reduced avoidance behavior. **a** Path plots showing movement during vehicle and SalB treatment on zero-maze. **b** Time in open arms, **c** movements into open arms, **d** movements into the closed arms, **e** correlation between time in open arms and number of movements into the open. **f**–**j** Same data format as (**a**–**e**), but for open field. Black lines reflect paired comparison for individual mice, red lines are linear regressions. *’s indicate significance with paired 1-tailed *t*-tests

Finally, we predicted that inhibiting iMSNs would reduce avoidance in these tasks. We inhibited the output of iMSNs with a kappa-opiate receptor (KOR)-based DREADD, a Gi-coupled receptor that is activated by systemic administration of the ligand Salvinorin B (SalB [54]). We expressed this receptor in iMSNs of A2A-cre mice (histology in Figure S9), and examined their behavior on the zero-maze and open field in a counter-balanced design, in which each mouse received SalB or vehicle on separate days, one week apart. Consistent with our prediction, SalB increased the time spent in the open arms and center (Fig. 5a,b,f,g, one-tailed paired *t*-test: both *p* < 0.05, full statistics in table S1). DREADD mediated inhibition of iMSNs selectively increased the number of movements into these areas (Fig. 5c,h, one-tailed paired *t*-test: both *p* < 0.05). Importantly, these effects occurred without altering the number of movements into the closed arms or surround (Fig. 5d,i, full statistics in table S1). Across individuals, the number of movements into the center was again strongly correlated with the total time in the open arms and center of the open field (Fig. 5e,j, both *R*^2^ > 0.66, *p* < 0.0001). In analyses of finer aspects of movement, we found that DREADD activation did not alter average velocity, velocity of movements into the open or closed areas of the task, nor average duration of movement (Figure S7). In this way, inhibiting iMSN output reduced avoidance in both tasks wholly independent of changes in other aspects of movement.

We also tested whether this manipulation could rescue the heightened avoidance behavior of iMSN-Drd2KO mice. The KOR DREADD was expressed in a new group of iMSN-Drd2KO mice (*n* = 6), and they were tested under identical conditions as above. Relative to vehicle, SalB caused a trend towards an increase in time spent in the open arms (Figure S8A, one-tailed *t*-test, *p* = 0.08), and significantly increased the time in center of the open field (Figure S8G, *p* < 0.01). This was also associated with a significant increase in average velocity in the zero maze (*p* < 0.05) but not open field (Figure S8D, J). while also increasing the duration of movements (Figure S6J, *p* < 0.05). Trends were noted for increased numbers of movements into the center (Figure S6H, *p* = 0.07) and decreased numbers of movements into the surround in the open field (Figure S6K, *p* = 0.07), but not the zero maze. Finally, no change was noted for the velocity of movements into the open or closed areas of these tasks (Figure S6E, F, K, L). These results suggest that inhibition of iMSN activity in iMSN-Drd2KO mice can rescue some of the heightened avoidance behavior, although it is associated with other changes in movement in these mice.

## Discussion

While the striatum has been implicated in resolving approach-avoidance conflicts, its precise role in this process remains unclear. Due to the role of striatal iMSNs in controlling both aversion [36] and the inhibition of actions [51], we hypothesized that iMSNs may serve to bias decision-making towards avoidance under conditions of uncertainty or danger. This would allow more time for information to accrue before an animal selects an action. Here, we report several experimental results that are consistent with this hypothesis. Removing D2Rs from iMSNs (but not dopaminergic or cholinergic neurons) decreased the time mice spent in the open areas of the zero maze and the open field, which was linked to reductions in movements into these areas. Population calcium recordings revealed disorganized iMSN activity around these movements in mice lacking D2Rs on iMSNs. Disrupting the output of iMSNs with low levels of optogenetic stimulation also enhanced avoidance, while inhibition of iMSNs via DREADD receptors reduced it. While our experiments characterized acute effects of manipulating iMSN output, plasticity in these circuits [56, 57] may lead to chronic elevations in avoidance in disorders such as social anxiety disorder or agoraphobia.

Across all experiments, the strongest behavioral changes involved reductions in the number of movements initiated into open areas, a measure of avoidance. Due to the role of iMSNs in controlling movement, we carefully considered the impact of alterations in global movement levels on our results. Interestingly, none of our manipulations modified the number of movements that mice made into the closed arms or surround of an open field, indicating that animals were able to initiate movements into “safe” areas of these tasks with normal frequency. Across all manipulations, mice also exhibited similar speeds while moving as control mice, again highlighting that manipulations of iMSNs mainly interfere with the initiation, and not execution of movement. That said, we did observe a decreased duration of movements in several manipulations, also consistent with previous studies [50]. Short, small movements are also consistent with risk assessment behavior [3, 4] and moving under approach-avoidance conflict, in which animals “dither between approach and avoidance” when at the balance point between the two [8].

It has been reported that over-expression of D2R in striatal iMSNs also disrupt behavioral flexibility and action selection [58]. Combining this with our present results suggests that perturbations in D2R expression in striatal MSNs disrupt action selection and initiation, regardless of whether expression is increased or decreased. While it is possible that some of the behavioral effects we observed could also be due to alterations in D2R expression during development, we do not think our main results depend on development. First, we were able to modulate the behavior of these mice via virally expressed opsins and DREADDs in adult wildtype animals. In addition, Cre-mediated knockdown of the D2R in iMSNs was sufficient to induce bradykinesia in adult mice [50].

Though prior work demonstrated reductions in action potential rates of MSNs in awake iMSN-Drd2KO mice [50], it was difficult to assess this point in our fiber photometry recordings. Overall fluorescence levels in fiber photometry recordings can be sensitive to the expression levels of gCaMP6 as well as the placement of the optical implant relative to the infection site, factors that can vary between animals. As such, fiber photometry signals are typically normalized to total fluorescence [53], as we did here. In addition, calcium recordings via fiber photometry can be sensitive to sub-threshold events as well as action potentials, whereas extracellular electrophysiology is only sensitive to action potentials. For these reasons, we were unable to directly compare our findings to those from Lemos et al. [50], and assess whether overall levels of iMSN activity were reduced in our fiber photometry recordings. However, consistent with electrophysiological recordings of these neurons, we observed a disorganization in iMSN output in iMSN-Drd2KO mice. Prior reports have noted enhanced GABA release from iMSNs in iMSN-Drd2KO mice [50] and alterations in striatal lateral inhibition [59], both of which may be responsible for the disruption of movement-related calcium activity we observed here.

In a causal test of this idea, we found that tonic stimulation of iMSNs, or brief optogenetic stimulation as mice approached the open arm, increased avoidance behavior. We interpret these optogenetic experiments as artificially de-coupling iMSN output from current behavioral processes. In this way, optogenetic disruption of iMSN activity may be similar to the tonic enhancements in GABA output observed in iMSN-Drd2KO mice [50]. Consistently, reducing iMSN output via DREADD-mediated inhibition of iMSNs increased approaches into, and time spent in, the open areas of both tasks. These effects occurred in the absence of more global changes in movement, a promising profile for potential therapeutics. Many anxiety disorders are characterized by high levels of passive avoidance (i.e., agoraphobia and social anxiety disorder). In addition, conflict itself can act as a cost, which can boost learning from punishment [60]. In anxiety disorders, this may contribute to a negative cycle, whereby a conflicting experience can increase the anxiogenic properties of similar experiences in the future. While multiple classes of medications are currently prescribed to treat anxiety (benzodiazepines, barbiturates, opiates, anti-depressants), those that primarily engage the dopamine system are notably absent [61]. This is due to concerns about both abuse liability and the potential of dopaminergic manipulations to exacerbate anxiety. While these are concerns for medications that non-selectively engage the dopamine system, targeting specific dopaminergic *sub-circuits* may modify anxiety states without such effects. The D2/D3 agonist ropinirole has been shown to have promising anxiolytic effects without producing global changes in movement [62–64], although the population of D2Rs mediating the anxiolytic actions is not known. Other potential mechanisms for modulating activity of iMSNs may include modulating adenosine-2A receptors, which has shown potential in pre-clinical tests of depression but so far not anxiety [65–67]. While more work is needed, therapies that inhibit the output of iMSNs may be a promising approach for reducing avoidance behavior in anxiety disorders.

## Electronic supplementary material

Figure S1

Figure S2

Figure S3

Figure S4

Figure S5

Figure S6

Figure S7

Figure S8

Figure S9

Supplemental Figure Captions

Table S1

## References

[1] McNaughton N, Gray JA (2000). Anxiolytic action on the behavioural inhibition system implies multiple types of arousal contribute to anxiety. J Affect Disord.

[2] Corr PJ, DeYoung CG, McNaughton N. Motivation and personality: a neuropsychological perspective. *Social and Personality Psychology Compas*s 2013; **7**:158–75.

[3] Blanchard DC, Griebel G, Pobbe R, Blanchard RJ (2011). Risk assessment as an evolved threat detection and analysis process. Neurosci Biobehav Rev.

[4] Bach DR (2015). Anxiety-like behavioural inhibition is normative under environmental threat-reward correlations. PLoS Comput Biol.

[5] Gray JA (1978). The neuropsychology of anxiety. Br J Psychol.

[6] Cryan JF, Sweeney FF (2011). The age of anxiety: role of animal models of anxiolytic action in drug discovery. Br J Pharmacol.

[7] Kirlic N, Young J, Aupperle RL (2017). Animal to human translational paradigms relevant for approach avoidance conflict decision making. Behav Res Ther.

[8] McNaughton N, DeYoung CG, Corr PJ, Absher JR, Cloutier J (2016). Approach/avoidance. Neuroimaging personality, social cognition, and character.

[9] Voorn P, Vanderschuren LJ, Groenewegen HJ, Robbins TW, Pennartz CM (2004). Putting a spin on the dorsal-ventral divide of the striatum. Trends Neurosci.

[10] Wall NR, De La Parra M, Callaway EM, Kreitzer AC (2013). Differential innervation of direct- and indirect-pathway striatal projection neurons. Neuron.

[11] Hunnicutt BJ, Jongbloets BC, Birdsong WT, Gertz KJ, Zhong H, Mao T. A comprehensive excitatory input map of the striatum reveals novel functional organization. *Elife* 2016;5:e19103. 10.7554/eLife.19103 PMC5207773 27892854

[12] Mogenson GJ, Swanson LW, Wu M (1983). Neural projections from nucleus accumbens to globus pallidus, substantia innominata, and lateral preoptic-lateral hypothalamic area: an anatomical and electrophysiological investigation in the rat. J Neurosci.

[13] Groenewegen HJ, Russchen FT (1984). Organization of the efferent projections of the nucleus accumbens to pallidal, hypothalamic, and mesencephalic structures: a tracing and immunohistochemical study in the cat. J Comp Neurol.

[14] Janak PH, Tye KM (2015). From circuits to behaviour in the amygdala. Nature.

[15] Nieh EH, Kim SY, Namburi P, Tye KM (2013). Optogenetic dissection of neural circuits underlying emotional valence and motivated behaviors. Brain Res.

[16] Parkinson JA, Olmstead MC, Burns LH, Robbins TW, Everitt BJ (1999). Dissociation in effects of lesions of the nucleus accumbens core and shell on appetitive pavlovian approach behavior and the potentiation of conditioned reinforcement and locomotor activity by D-amphetamine. J Neurosci.

[17] Parkinson JA, Dalley JW, Cardinal RN, Bamford A, Fehnert B, Lachenal G (2002). Nucleus accumbens dopamine depletion impairs both acquisition and performance of appetitive Pavlovian approach behaviour: implications for mesoaccumbens dopamine function. Behav Brain Res.

[18] Contreras-Vidal JL, Schultz W (1999). A predictive reinforcement model of dopamine neurons for learning approach behavior. J Comput Neurosci.

[19] Di Ciano P, Cardinal RN, Cowell RA, Little SJ, Everitt BJ (2001). Differential involvement of NMDA, AMPA/kainate, and dopamine receptors in the nucleus accumbens core in the acquisition and performance of pavlovian approach behavior. J Neurosci.

[20] Tye KM, Mirzabekov JJ, Warden MR, Ferenczi EA, Tsai HC, Finkelstein J (2013). Dopamine neurons modulate neural encoding and expression of depression-related behaviour. Nature.

[21] Francis TC, Chandra R, Friend DM, Finkel E, Dayrit G, Miranda J (2015). Nucleus accumbens medium spiny neuron subtypes mediate depression-related outcomes to social defeat stress. Biol Psychiatry.

[22] Al-Hasani R, McCall JG, Shin G, Gomez AM, Schmitz GP, Bernardi JM (2015). Distinct subpopulations of nucleus accumbens dynorphin neurons drive aversion and reward. Neuron.

[23] Salamone JD (1994). The involvement of nucleus accumbens dopamine in appetitive and aversive motivation. Behav Brain Res.

[24] Bromberg-Martin ES, Matsumoto M, Hikosaka O (2010). Dopamine in motivational control: rewarding, aversive, and alerting. Neuron.

[25] Green RH, Beatty WW, Schwartzbaum JS (1967). Comparative effects of septo-hippocampal and caudate lesions on avoidance behavior in rats. J Comp Physiol Psychol.

[26] Winocur G, Mills JA (1969). Effects of caudate lesions on avoidance behavior in rats. J Comp Physiol Psychol.

[27] Allen JD, Davison CS (1973). Effects of caudate lesions on signaled and nonsignaled Sidman avoidance in the rat. Behav Biol.

[28] Prado-Alcala RA, Grinberg ZJ, Arditti ZL, Garcia MM, Prieto HG, Brust-Carmona H (1975). Learning deficits produced by chronic and reversible lesions of the corpus striatum in rats. Physiol Behav.

[29] Rothman AH, Glick SD (1976). Differential effects of unilateral and bilateral caudate lesions on side preference and passive avoidance behavior in rats. Brain Res.

[30] Aupperle RL, Paulus MP (2010). Neural systems underlying approach and avoidance in anxiety disorders. Dialog- Clin Neurosci.

[31] Friedman A, Homma D, Gibb LG, Amemori K, Rubin SJ, Hood AS (2015). A corticostriatal path targeting striosomes controls decision-making under conflict. Cell.

[32] Sugam JA, Saddoris MP, Carelli RM (2014). Nucleus accumbens neurons track behavioral preferences and reward outcomes during risky decision making. Biol Psychiatry.

[33] Aupperle RL, Melrose AJ, Francisco A, Paulus MP, Stein MB (2015). Neural substrates of approach-avoidance conflict decision-making. Hum Brain Mapp.

[34] Gerfen CR, Engber TM, Mahan LC, Susel Z, Chase TN, Monsma FJ (1990). D1 and D2 dopamine receptor-regulated gene expression of striatonigral and striatopallidal neurons. Science.

[35] Hikida T, Kimura K, Wada N, Funabiki K, Nakanishi S (2010). Distinct roles of synaptic transmission in direct and indirect striatal pathways to reward and aversive behavior. Neuron.

[36] Kravitz AV, Tye LD, Kreitzer AC (2012). Distinct roles for direct and indirect pathway striatal neurons in reinforcement. Nat Neurosci.

[37] Freeze BS, Kravitz AV, Hammack N, Berke JD, Kreitzer AC (2013). Control of basal ganglia output by direct and indirect pathway projection neurons. J Neurosci.

[38] Zalocusky KA, Ramakrishnan C, Lerner TN, Davidson TJ, Knutson B, Deisseroth K (2016). Nucleus accumbens D2R cells signal prior outcomes and control risky decision-making. Nature.

[39] Tecuapetla F, Jin X, Lima SQ, Costa RM (2016). Complementary contributions of striatal projection pathways to action initiation and execution. Cell.

[40] Schneier FR, Liebowitz MR, Abi-Dargham A, Zea-Ponce Y, Lin SH, Laruelle M (2000). Low dopamine D(2) receptor binding potential in social phobia. Am J Psychiatry.

[41] Lawford BR, Young R, Noble EP, Kann B, Ritchie T (2006). The D2 dopamine receptor (DRD2) gene is associated with co-morbid depression, anxiety and social dysfunction in untreated veterans with post-traumatic stress disorder. Eur Psychiatry.

[42] Joe KH, Kim DJ, Park BL, Yoon S, Lee HK, Kim TS (2008). Genetic association of DRD2 polymorphisms with anxiety scores among alcohol-dependent patients. Biochem Biophys Res Commun.

[43] Bailer UF, Frank GK, Price JC, Meltzer CC, Becker C, Mathis CA (2013). Interaction between serotonin transporter and dopamine D2/D3 receptor radioligand measures is associated with harm avoidant symptoms in anorexia and bulimia nervosa. Psychiatry Res.

[44] Frank MJ, Hutchison K (2009). Genetic contributions to avoidance-based decisions: striatal D2 receptor polymorphisms. Neuroscience.

[45] Bello EP, Mateo Y, Gelman DM, Noain D, Shin JH, Low MJ (2011). Cocaine supersensitivity and enhanced motivation for reward in mice lacking dopamine D2 autoreceptors. Nat Neurosci.

[46] Delle Donne KT, Sesack SR, Pickel VM (1996). Ultrastructural immunocytochemical localization of neurotensin and the dopamine D2 receptor in the rat nucleus accumbens. J Comp Neurol.

[47] Li X, Qi J, Yamaguchi T, Wang HL, Morales M (2013). Heterogeneous composition of dopamine neurons of the rat A10 region: molecular evidence for diverse signaling properties. Brain Struct Funct.

[48] Sesack SR, Aoki C, Pickel VM (1994). Ultrastructural localization of D2 receptor-like immunoreactivity in midbrain dopamine neurons and their striatal targets. J Neurosci.

[49] Alcantara AA, Chen V, Herring BE, Mendenhall JM, Berlanga ML (2003). Localization of dopamine D2 receptors on cholinergic interneurons of the dorsal striatum and nucleus accumbens of the rat. Brain Res.

[50] Lemos JC, Friend DM, Kaplan AR, Shin JH, Rubinstein M, Kravitz AV (2016). Enhanced GABA transmission drives bradykinesia following loss of dopamine D2 receptor signaling. Neuron.

[51] Kravitz AV, Freeze BS, Parker PR, Kay K, Thwin MT, Deisseroth K (2010). Regulation of parkinsonian motor behaviours by optogenetic control of basal ganglia circuitry. Nature.

[52] Balleine BW, Delgado MR, Hikosaka O (2007). The role of the dorsal striatum in reward and decision-making. J Neurosci.

[53] Cui G, Jun SB, Jin X, Pham MD, Vogel SS, Lovinger DM (2013). Concurrent activation of striatal direct and indirect pathways during action initiation. Nature.

[54] Vardy E, Robinson JE, Li C, Olsen RH, DiBerto JF, Giguere PM (2015). A new DREADD facilitates the multiplexed chemogenetic interrogation of behavior. Neuron.

[55] Chen TW, Wardill TJ, Sun Y, Pulver SR, Renninger SL, Baohan A (2013). Ultrasensitive fluorescent proteins for imaging neuronal activity. Nature.

[56] Kravitz AV, Kreitzer AC (2012). Striatal mechanisms underlying movement, reinforcement, and punishment. Physiology.

[57] Beeler JA, Frank MJ, McDaid J, Alexander E, Turkson S, Bernardez Sarria MS (2012). A role for dopamine-mediated learning in the pathophysiology and treatment of Parkinson's disease. Cell Rep.

[58] Drew MR, Simpson EH, Kellendonk C, Herzberg WG, Lipatova O, Fairhurst S (2007). Transient overexpression of striatal D2 receptors impairs operant motivation and interval timing. J Neurosci.

[59] Dobbs LK, Kaplan AR, Lemos JC, Matsui A, Rubinstein M, Alvarez VA (2016). Dopamine regulation of lateral inhibition between striatal neurons gates the stimulant actions of cocaine. Neuron.

[60] Cavanagh JF, Masters SE, Bath K, Frank MJ (2014). Conflict acts as an implicit cost in reinforcement learning. Nat Commun.

[61] Craske MG, Stein MB (2016). Anxiety. Lancet.

[62] Mavrikaki M, Schintu N, Nomikos GG, Panagis G, Svenningsson P (2014). Ropinirole regulates emotionality and neuronal activity markers in the limbic forebrain. Int J Neuropsychopharmacol.

[63] Rogers DC, Costall B, Domeney AM, Gerrard PA, Greener M, Kelly ME (2000). Anxiolytic profile of ropinirole in the rat, mouse and common marmoset. Psychopharmacology.

[64] Rektorova I, Balaz M, Svatova J, Zarubova K, Honig I, Dostal V (2008). Effects of ropinirole on nonmotor symptoms of Parkinson disease: a prospective multicenter study. Clin Neuropharmacol.

[65] Yamada K, Kobayashi M, Shiozaki S, Ohta T, Mori A, Jenner P (2014). Antidepressant activity of the adenosine A2A receptor antagonist, istradefylline (KW-6002) on learned helplessness in rats. Psychopharmacology.

[66] El Yacoubi M, Ledent C, Parmentier M, Bertorelli R, Ongini E, Costentin J (2001). Adenosine A2A receptor antagonists are potential antidepressants: evidence based on pharmacology and A2A receptor knockout mice. Br J Pharmacol.

[67] Yamada K, Kobayashi M, Kanda T (2014). Involvement of adenosine A2A receptors in depression and anxiety. Int Rev Neurobiol.

[68] Gerfen Charles R., Paletzki Ronald, Heintz Nathaniel (2013). GENSAT BAC Cre-Recombinase Driver Lines to Study the Functional Organization of Cerebral Cortical and Basal Ganglia Circuits. Neuron.

