# Correction: Striatopallidal neurons control avoidance behavior in exploratory tasks

**DOI:** 10.1038/s41380-019-0460-y

**Published:** 2019-07-31

**Authors:** Kimberly H. LeBlanc, Tanisha D. London, Ilona Szczot, Miriam E. Bocarsly, Danielle M. Friend, Katrina P. Nguyen, Marda M. Mengesha, Marcelo Rubinstein, Veronica A. Alvarez, Alexxai V. Kravitz

**Affiliations:** 1 National Institute of Diabetes and Digestive and Kidney Diseases, National Institutes of Health, Bethesda, MD 20892 USA; 2 National Institute on Alcohol Abuse and Alcoholism, National Institutes of Health, Bethesda, MD 20892 USA; 3 Instituto de Investigaciones en Ingeniería Genética y Biología Molecular, CONICET, Buenos Aires, C1428ADN Argentina; 4 FCEN, Universidad de Buenos Aires, Buenos Aires, C1428EGA Argentina; 5 Department of Molecular and Integrative Physiology, University of Michigan Medical School, Ann Arbor, MI 48109 USA; 6 National Institute on Drug Abuse, National Institutes of Health, Bethesda, MD 20892 USA

**Keywords:** Neuroscience, Physiology

**Correction to: Molecular Psychiatry**

10.1038/s41380-018-0051-3 published online 25 April 2018

This article was originally published under standard licence, but has now been made available under a [CC BY 4.0] license. The PDF and HTML versions of the paper have been modified accordingly.

