# Supplementary figures and images for: Striatopallidal neurons control avoidance behavior in exploratory tasks
Source: Mol Psychiatry. 2018 Apr 25;25(2):491–505. doi: 10.1038/s41380-018-0051-3 (PMC6202282; doi:10.1038/s41380-018-0051-3)

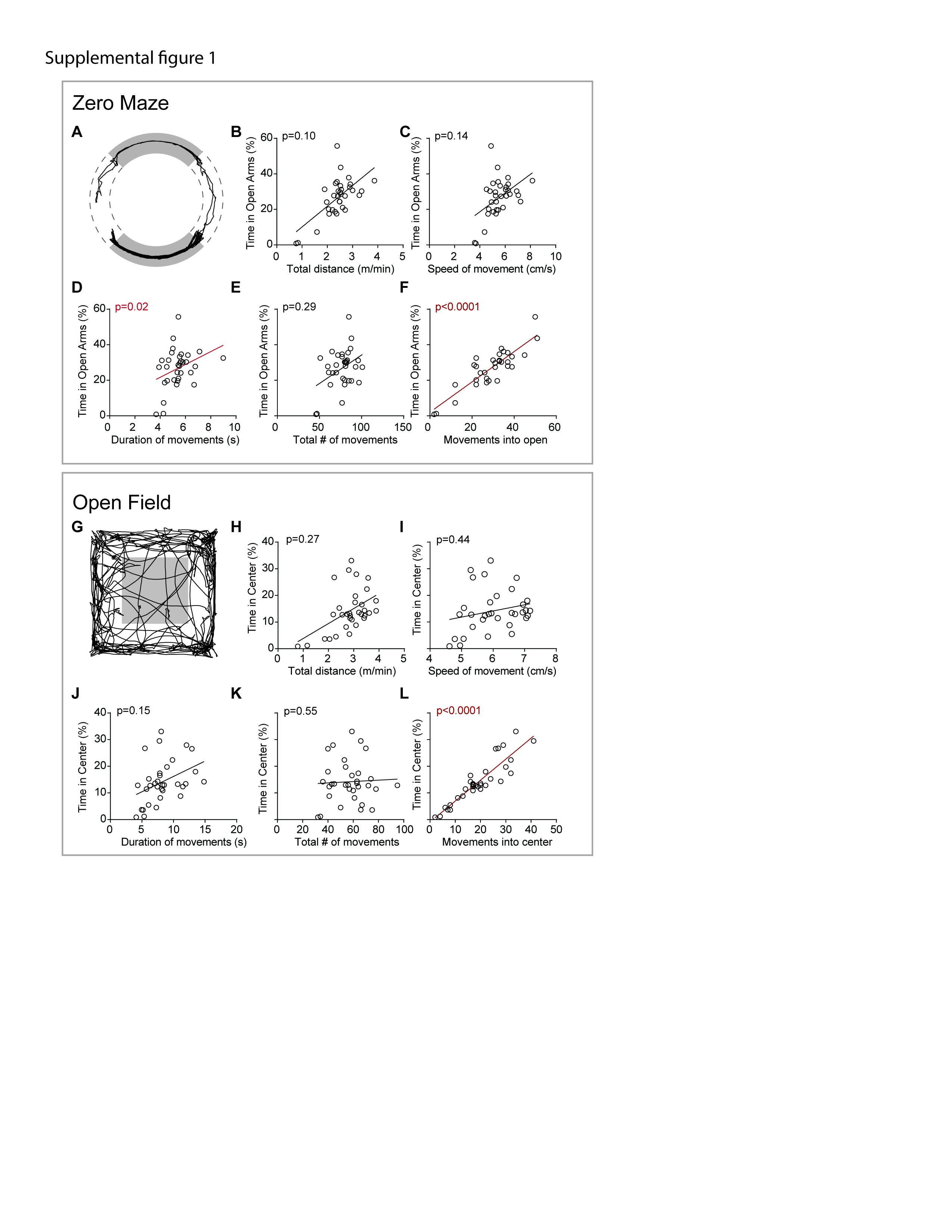

Supplement: Supplementary file 1 — Figure S1 [file 41380_2018_51_MOESM1_ESM.tif]

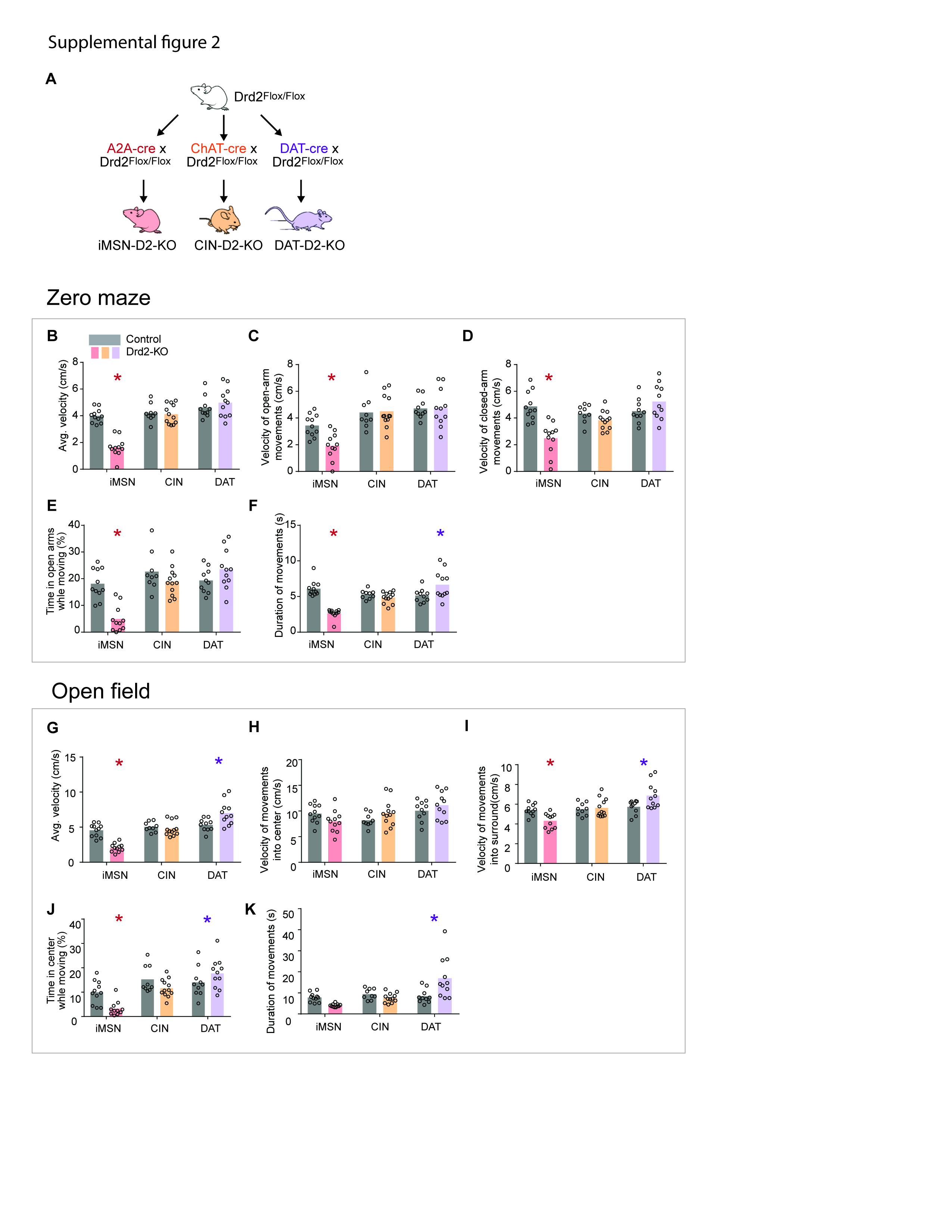

Supplement: Supplementary file 2 — Figure S2 [file 41380_2018_51_MOESM2_ESM.tif]

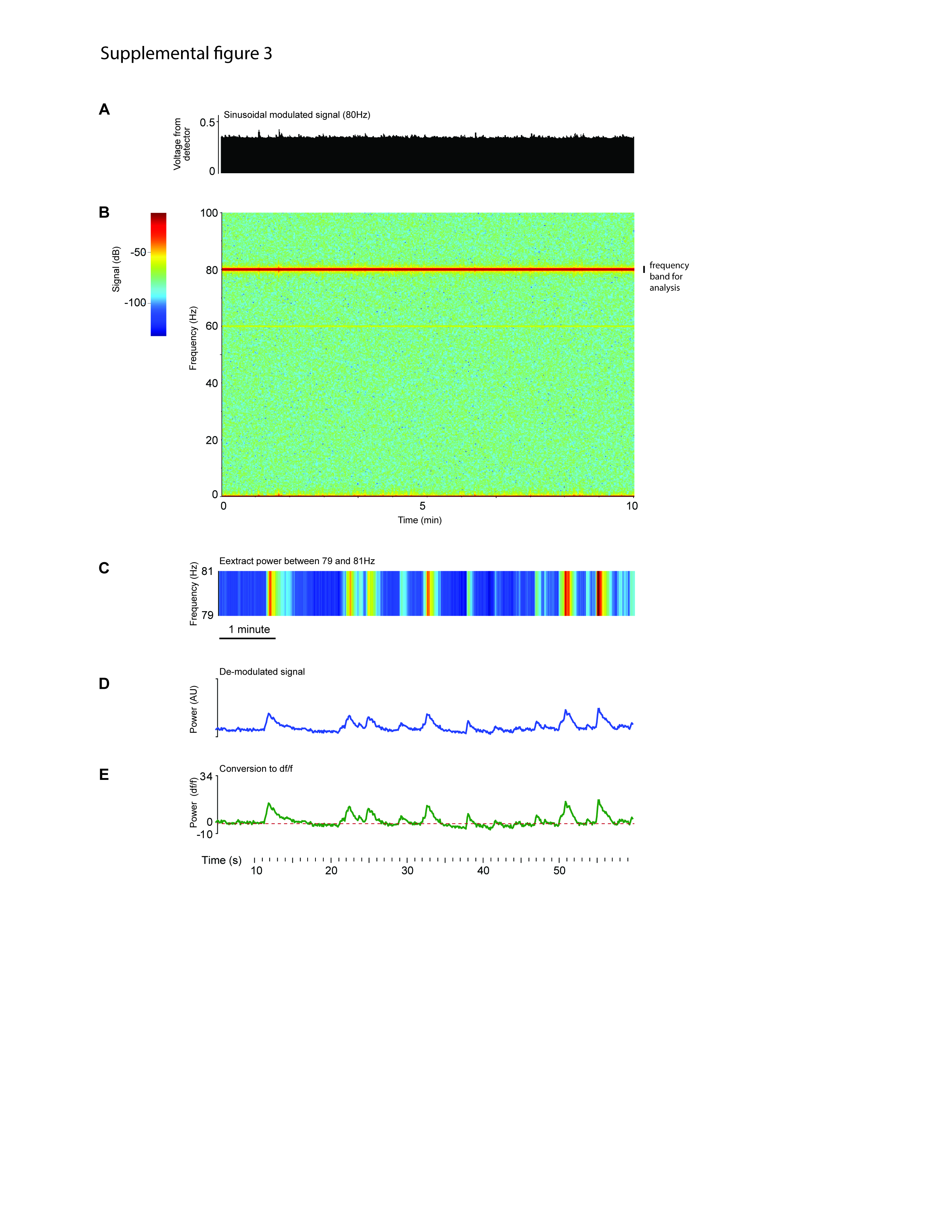

Supplement: Supplementary file 3 — Figure S3 [file 41380_2018_51_MOESM3_ESM.tif]

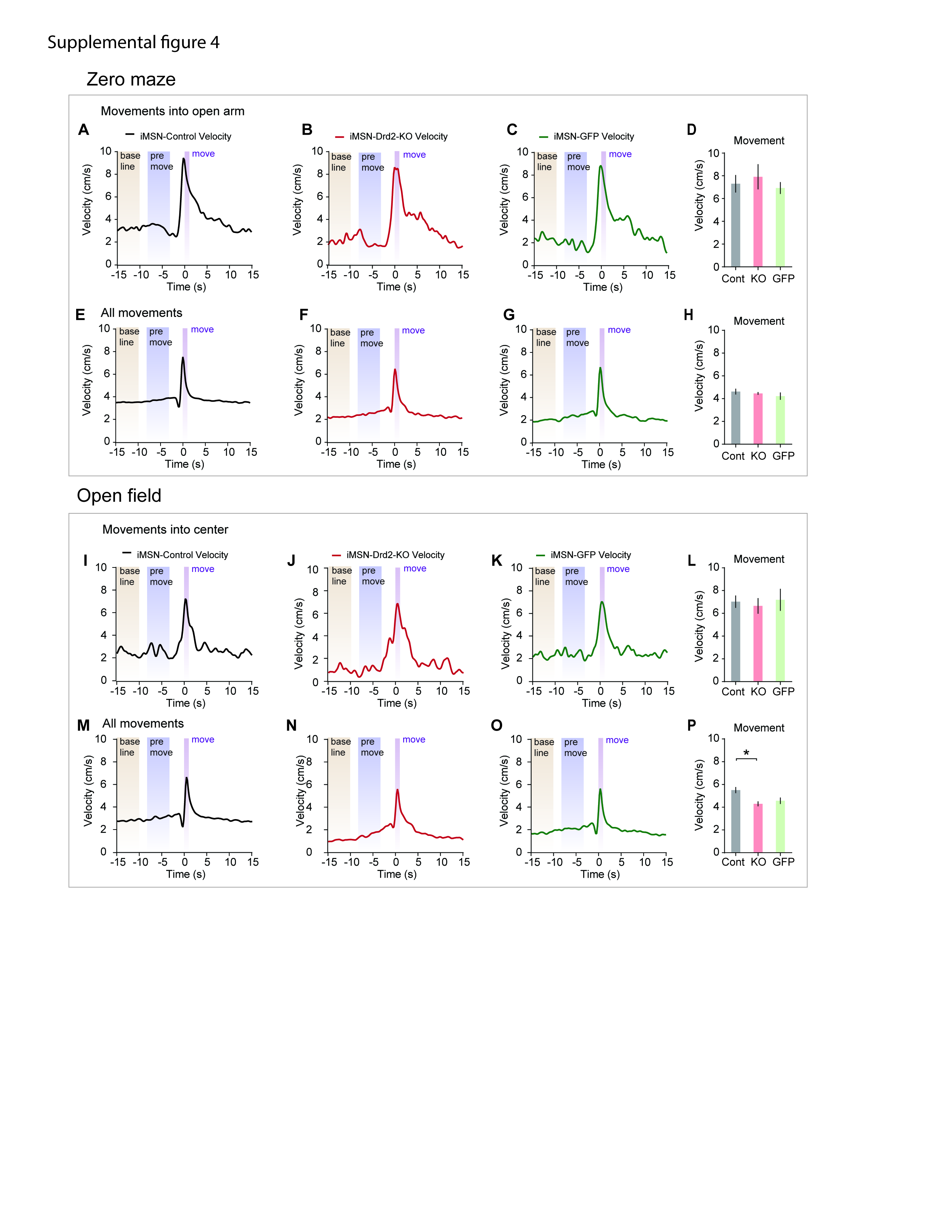

Supplement: Supplementary file 4 — Figure S4 [file 41380_2018_51_MOESM4_ESM.tif]

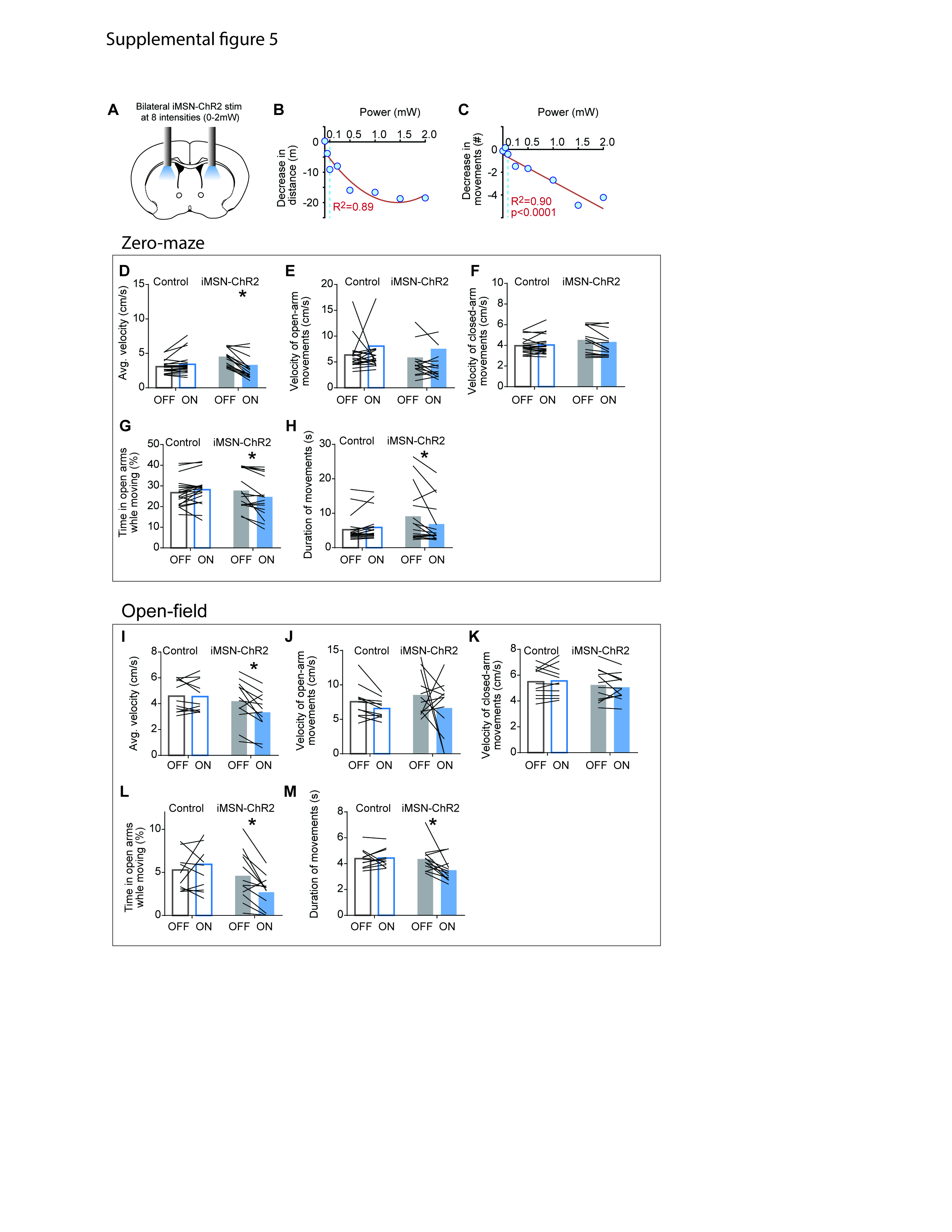

Supplement: Supplementary file 5 — Figure S5 [file 41380_2018_51_MOESM5_ESM.tif]

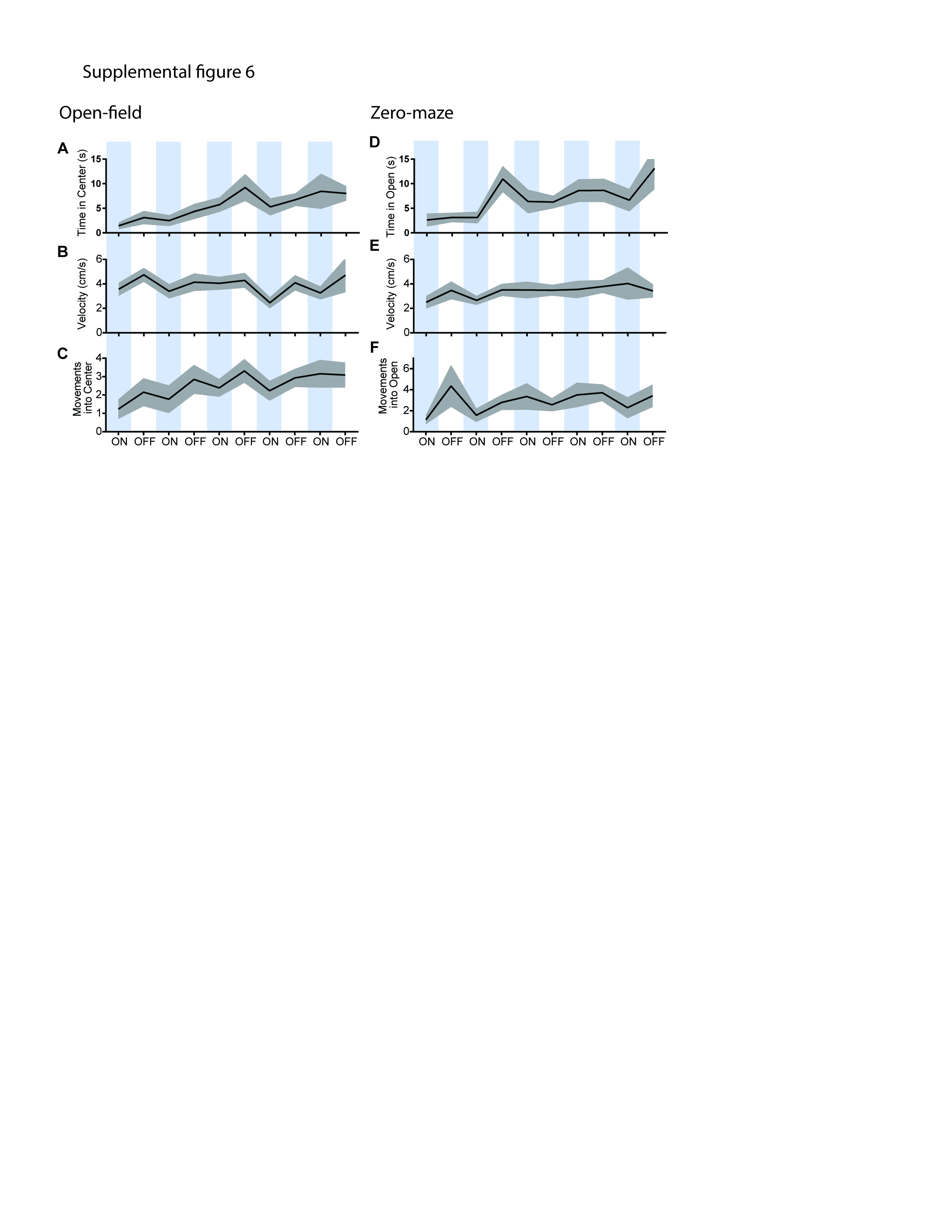

Supplement: Supplementary file 6 — Figure S6 [file 41380_2018_51_MOESM6_ESM.tif]

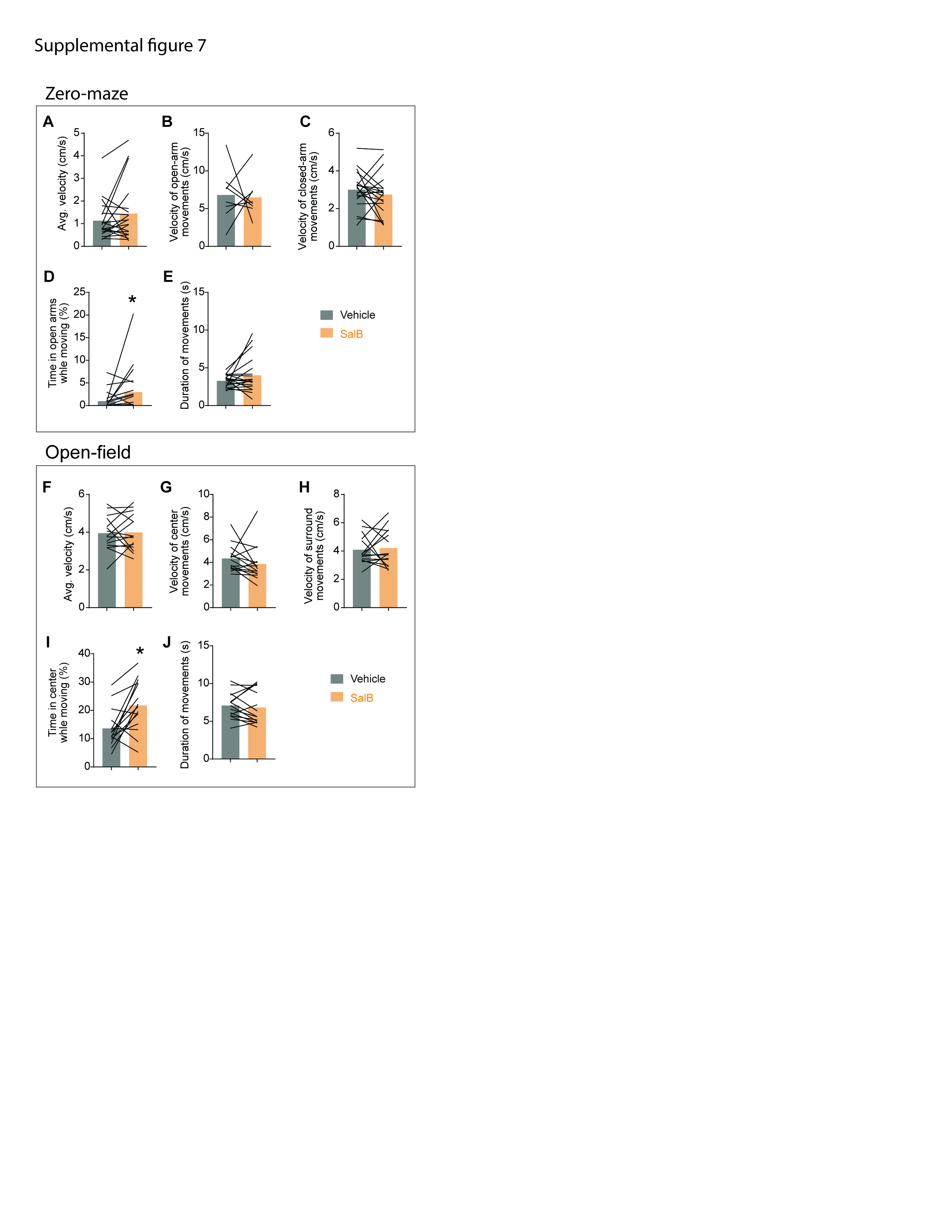

Supplement: Supplementary file 7 — Figure S7 [file 41380_2018_51_MOESM7_ESM.tif]

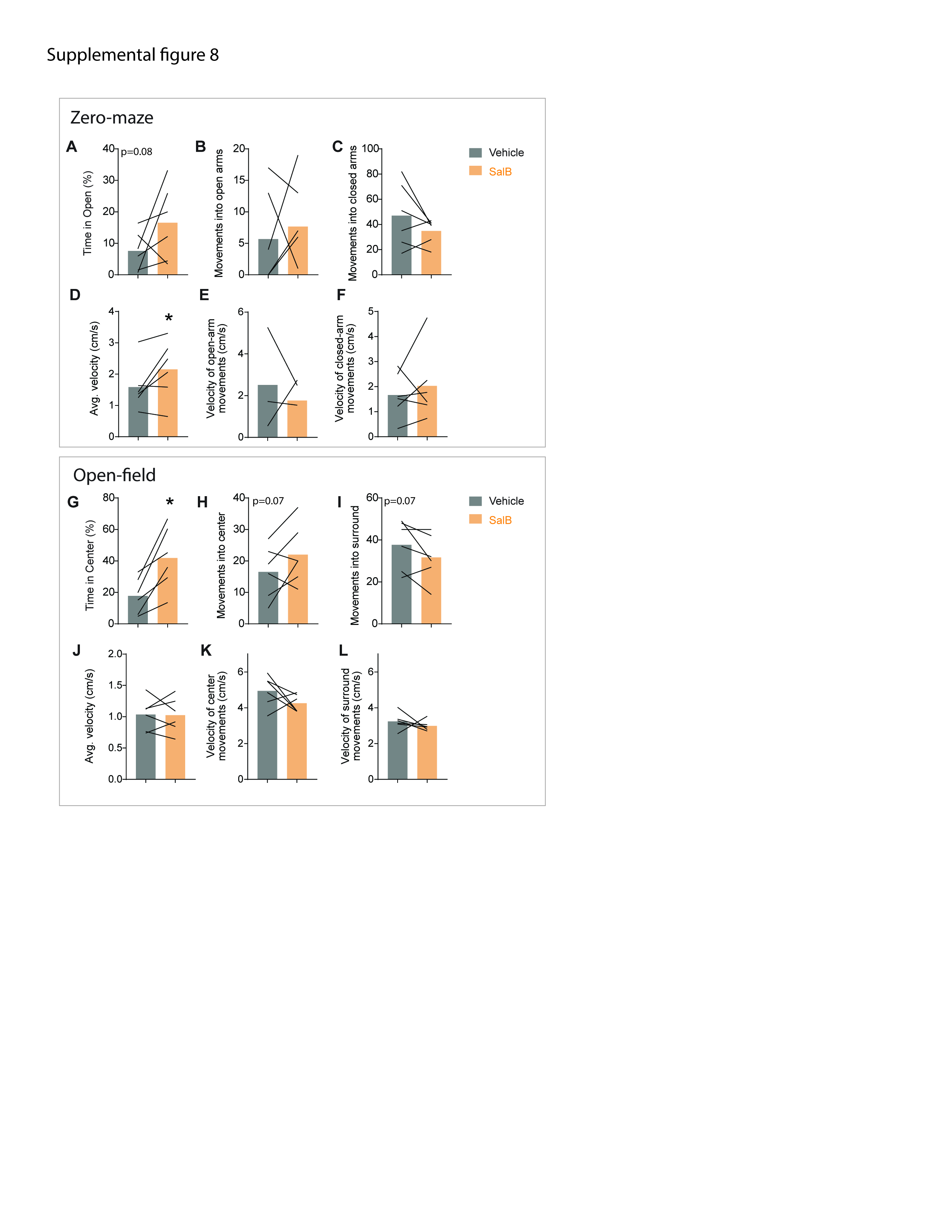

Supplement: Supplementary file 8 — Figure S8 [file 41380_2018_51_MOESM8_ESM.tif]

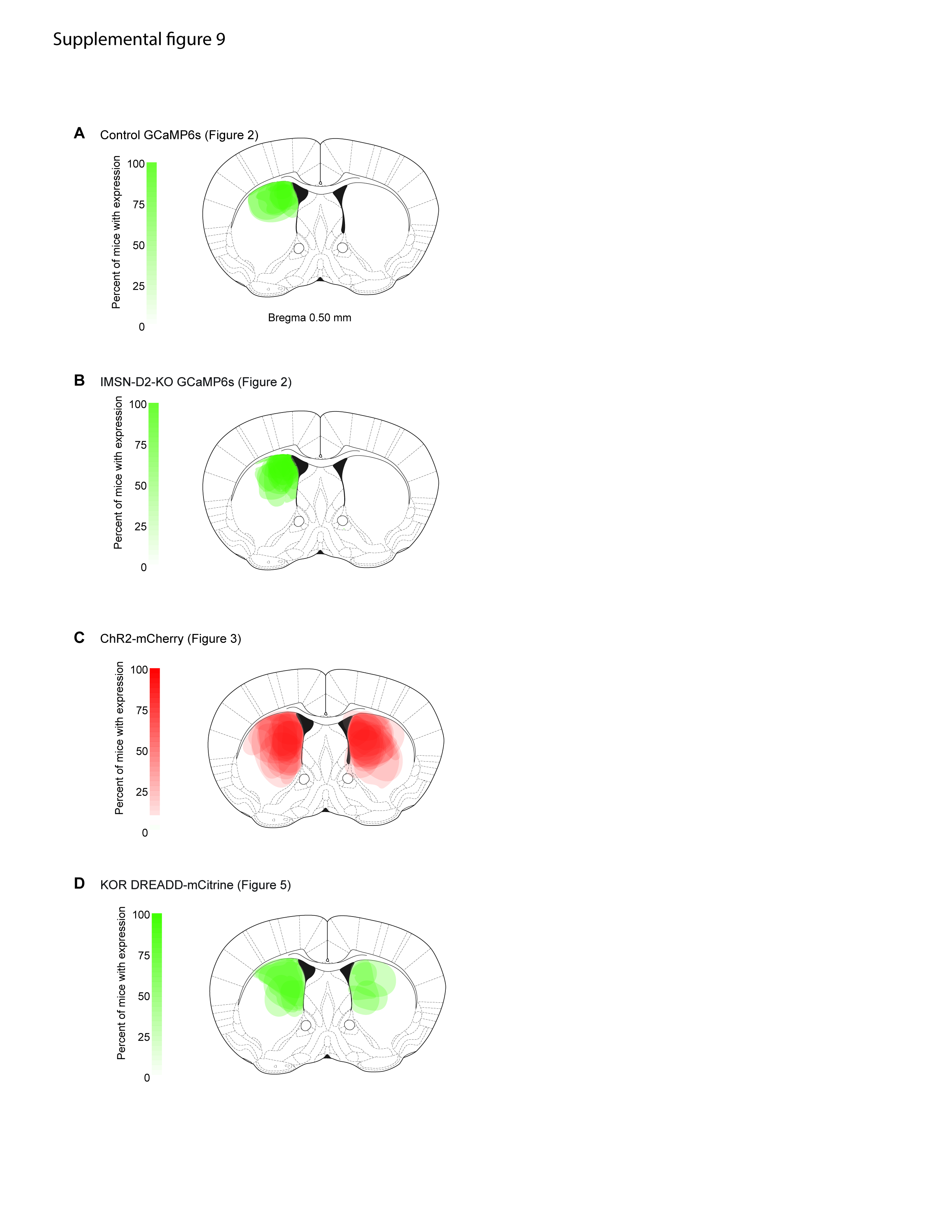

Supplement: Supplementary file 9 — Figure S9 [file 41380_2018_51_MOESM9_ESM.tif]
