# Supplementary material for: Striatopallidal neurons control avoidance behavior in exploratory tasks
Source: Mol Psychiatry. 2018 Apr 25;25(2):491–505. doi: 10.1038/s41380-018-0051-3 (PMC6202282; doi:10.1038/s41380-018-0051-3)
Supplement: Supplementary file 10 — Supplemental Figure Captions [file 41380_2018_51_MOESM10_ESM.docx]

**Supplemental Figure Captions**

**Supplemental Figure 1: Behavioral correlates of avoidance behavior. A.** Path plot of zero maze behavior of a wildtype mouse. Multiple regression analysis between time in open arms (%) and **(B)** total distance moved**, (C)** speed of movement**, (D)** duration of movements**, (E)** total number of movements**,** and **(F)** number of movements into the open arms**. G-L.** Same format as **A-F**, but for time in the center of the open field. Points reflect individual mice, lines are linear regression fits.

**Supplemental Figure 2. Behavioral consequences of D2R removal from iMSNs, CINs, and DAT neurons. A.** Breeding scheme to generate iMSN-Drd2KO, CIN-Drd2KO, and DAT-Drd2KO mice. For zero maze, **(B)** Average velocity, **(C)** Velocity of open-arm movements, **(D)** Velocity of closed-arm movements, **(E)** Time in open while moving, and **(F)** Duration of movements. Data shown for all three mouse lines. **G-K**. Same format as **B-F**, but for open field. Points reflect individual mice. *s denote significance between Control and Drd2KO mice by Sidak’s post-hoc test following 2-way ANOVA.

**Supplemental figure 3.** **Photometry data processing work-flow.** **A**. 10 minutes of modulated data, **(B)** power spectra of the data in **(A)** shown from 0-100Hz, **(C)** power in 80Hz frequency band, **(D)** demodulated signal, and **(E)** transformation to df/f.

**Supplemental figure 4.** **Velocity** **of movements during** **photometry experiments.** Velocity of movements into the open arm of the zero maze for **(A)** iMSN-Control mice, **(B)** iMSN-Drd2KO mice, and **(C)** iMSN-GFP mice. **D.** Average df/f during baseline, pre-movement, and movement periods from **A-C**. **(E-H)** Same as **A-D** but for all movements on zero maze. **(I-P)** Same data presentation as **A-H** but for open field. * denotes significance between control and iMSN-Drd2KO by Sidak’s post-hoc test following 2-way ANOVA.

**Supplemental Figure 5.** **Effects of low power stimulation of iMSNs. A.** Schematic showing bilateral fiber placements in dorsomedial striatum. Difference between LED ON vs. OFF at 8 stimulation intensities for **(B)** distance moved and **(C)** number of movements. For zero maze, **(D)** Average velocity, **(E)** Velocity of open-arm movements, **(F)** Velocity of closed-arm movements, **(G)** Time in open while moving, and **(H)** Duration of movements. **I-M**. Same data presentation as **D-H** but for open field. Black lines reflect paired comparison for individual mice, *s denote significance between LED on vs. off conditions by Sidak’s post-hoc test following 2-way ANOVA.

**Supplemental Figure 6.** **Minute-by-minute analyses of optogenetic stimulation experiments.** **(A)** Time in center **(B)** average velocity, and **(C)** movements into center of open field. **(D-F)** Same as **A-C** but for zero maze. Blue bars indicate stimulation periods. Data presented as mean +/- SEM.

**Supplemental Figure 7. Effect of iMSN-KOR-DREADD activation on movement.** For zero maze, **(A)** Average velocity, **(B)** Velocity of open-arm movements, **(C)** Velocity of closed-arm movements, **(D)** Time in open while moving, and **(E)** Duration of movements. **F-J**. Same data presentation as **A-E** but for open field. Black lines reflect paired comparison for individual mice. *’s indicate significance with paired 1-tailed t-tests.

**Supplemental Figure 8. Effect of iMSN-KOR-DREADD stimulation in iMSN-Drd2KO mice. (A)** Time in open arms, **(B)** Movements into the open arms, **(C)** Movements into the closed arms, **(D)** Average velocity, **(E)** Velocity of open-arm movements, **(F)** Velocity of closed-arm movements. **(G-L)**, Same data format as **A-F**, but for open field. Black lines reflect paired comparison for individual mice. *’s indicate significance with paired 1-tailed t-tests.

**Supplemental Figure 9.** **Histology for viral injections.** Shaded regions show viral expression area traced from photographs for **(A, B)** GCaMP6, **(C)** ChR2, and **(D)** KOR-DREADD animals.

**Supplemental Table S1.** Complete statistical results for data in all figures.
